# Addition of Anionic Polysaccharide Stabilizers Modulates In Vitro Digestive Proteolysis of a Chocolate Milk Drink in Adults and Children

**DOI:** 10.3390/foods9091253

**Published:** 2020-09-07

**Authors:** Shlomit David, Maya Magram Klaiman, Avi Shpigelman, Uri Lesmes

**Affiliations:** Faculty of Biotechnology and Food Engineering, Technion-Israel Institute of Technology, Haifa 3200003, Israel; shlomit2@campus.technion.ac.il (S.D.); mayamag18@gmail.com (M.M.K.); avis@bfe.technion.ac.il (A.S.)

**Keywords:** carrageenan, xanthan gum, stabilizers, chocolate milk drink, in vitro digestion, digestive proteolysis, bioactive peptides, bioaccessibility

## Abstract

There is a need to better understand the possible anti-nutritional effect of food stabilizers on the digestibility of important macronutrients, like proteins. This study hypothesized that the anionic nature of κ-, ι-, λ-, Carrageenan (CGN) and xanthan gum directs their interactions with food proteins leading to their subsequent attenuated digestive proteolysis. Model chocolate milk drinks were tested for their colloidal properties, viscosity and proteolytic breakdown in adults and children using in vitro digestion models coupled with proteomic analyses. SDS-PAGE analyses of gastro-intestinal effluents highlight stabilizers hinder protein breakdown in adults and children. Zeta potential and colloidal particle size were the strongest determinants of stabilizers’ ability to hinder proteolysis. LC-MS proteomic analyses revealed stabilizer addition significantly reduced bioaccessibility of milk-derived bioactive peptides with differences in liberated peptide sequences arising mainly from their location on the outer rim of the protein structures. Further, liberation of bioactive peptides emptying from a child stomach into the intestine were most affected by the presence of ι-CGN. Overall, this study raises the notion that stabilizer charge and other properties of edible proteins are detrimental to the ability of humans to utilize the nutritional potential of such formulations. This could help food professionals and regulatory agencies carefully consider the use of anionic stabilizers in products aiming to serve as protein sources for children and other liable populations.

## 1. Introduction

Food additives are widely used for a myriad of techno-functional purposes and are regulated by various agencies around the globe. Anionic polysaccharides from natural sources are central food additives that exhibit high functionality and high consumer acceptance. Such natural biopolymers, e.g., alginate, xanthan gum, carrageenan, starch and pectin, are widely used to manipulate food properties via various mechanisms, including macromolecular biopolymer interactions. For example, electrostatic biopolymer interactions between high methoxyl, low methoxyl pectin, xanthan gum, alginate or carrageenan with food proteins (such as whey proteins) govern their complexation and colloidal properties [1,2,3,4,5,6,7] In fact, harnessing polysaccharide-protein interactions to tailor food formulation properties, e.g., particulation and gelation, has been at the heart of numerous studies and reviews [8,9,10,11]. However, the implications of hydrocolloid interactions on the digestive fate of foods is not fully resolved and some argue food additives may attenuate digestion and compromise gut health [12,13,14,15].

This study is focused on carrageenan (CGN; E-407) and xanthan gum (XG; E-415), which are extensively used to shape the flow behavior and textural properties of a large variety of food products [16,17,18,19]. The first is a family of sulphated galactans produced from algae [20] while the latter is an exopolysaccharide produced during fermentation by *Xanthomonas campestris* [16]. Both those anionic polysaccharides are approved for food applications [17,19] and are documented to interact with dairy proteins [6,21,22]. The main mode of action is electrostatic biopolymer interactions [21,23], although physical entrapment and gelation have also been reported [24]. In fact, the rising use of food additives, such as anionic polysaccharide stabilizers and emulsifiers, has given rise to considerable debate on their possible ramifications to food’s digestive fate and overall consumer health [17,19,25]. For example, CGN safety has been under vivid public and professional debate [26,27].

In respect to the ramification of food stabilizers on food digestion, studies show carboxymethyl cellulose, pectin and guar gum may affect luminal mass transfer rates of simple carbohydrates [15,28]. Other studies show that CGN can hinder the breakdown of whey, soy and egg proteins and inhibit gastrointestinal proteases that ultimately attenuate the digestive fate of elemental macronutrients, such as proteins [13,29,30]. Such effects on digestive proteolysis may be further modulated by consumer gut physiology, which varies during the healthy lifespan [31,32,33,34,35]. Thus, there seems to be a gap in our understanding of the impact of anionic polysaccharide stabilizers on protein digestion in different age groups and liable populations with altered gastro-intestinal functions. For example, a recent study demonstrated that CGN can attenuate protein breakdown and hinder the bioaccessibility of whey-derived bioactive peptides in toddlers (David et al., 2020). Further elucidation of such effects in toddlers and children is in dire need, as they have been recently identified as a liable strata of the population with high levels of expected exposure to food stabilizers, such as CGN [36].

This research aimed to study whether and to which extent anionic polysaccharides may modulate the digestive proteolysis of a chocolate milk drink (CMD), as a model of a real product, consumed by various consumers but predominantly by children (two years old children or older). Three key commercial carrageenan preparations (κ-CGN, ι-CGN, λ-CGN) and xanthan gum were analyzed and used to stabilize test drinks. In turn, characterized test drinks were fed into semi-dynamic in vitro digestion models and effluents were analyzed by various proteomic analyses to gain insight into the bioaccessible peptide entities. Thus, enabling elucidation of the differential effects of the stabilizers on the proteolytic breakdown of milk proteins and the luminal bioaccessibility of bioactive peptides.

## 2. Materials and Methods

### 2.1. Materials

Food grade stabilizers and ingredients. Commercial κ-, ι- and λ-CGN (GenugelR type CHP-2, GenugelR type CJ and GenuviscoR type CSM-2, respectively) were provided by CP Kelco (Atlanta, GA, USA). Xanthan gum was kindly donated by Frutarom (Haifa, Israel). Sugar, 3% fat milk and cocoa powder were purchased from a local supermarket.

Reagents for in vitro digestion and subsequent analyses. Mucin from porcine stomach (cat. M2378, Lot 100M0187V); Pepsin from porcine gastric mucosa (cat. P7000, Lot BCBR4540V); Trypsin from porcine pancreas (cat. T0303, Lot SLBG6474V); α-chymotrypsin from bovine pancreas (cat. C4129, Lot SLBT5554); Taurocholic acid sodium salt hydrate (cat. T4009), Acrylamide/bis-acrylamide, 40% solution (cat. A7802) and phenylmethanesulfonylfluoride (PMSF) (cat. 93482) were purchased from Sigma-Aldrich (Rehovot, Israel). Sodium glycodeoxycholate (cat. GK3611) was purchased from Glentham Life Sciences (Corsham, UK). Comassie Brilliant Blue R250 was purchased from Bio-Rad (Rishon LeZion, Israel). Spectra Multicolor low range protein ladder (cat. 26628, Lot 00615745) and PageRuler Prestained Ladder (cat. 26616) were purchased from Thermo scientific. Simulated digestive fluids as well as bile were formulated following the INFOGEST harmonized protocol (Minekus et al., 2014). Age-related differences in the composition of these biofluids were made based on information gathered in relevant publications [31,33,37]. Double-distilled water (DDW) was used to make all samples and all other chemicals were of analytical grade.

### 2.2. Methods

#### 2.2.1. Characterization of Stabilizers

In house analyses were performed to characterize the food grade CGNs and XG.

Mineral content quantification. Since minerals are not just of nutritional importance but also affect electrostatic interactions all stabilizers were analyzed for mineral content. Solutions of κ-, ι- or λ- CGN and XG were prepared in DDW, pH 7, at room temperature at a final concentration of 1 mg/mL. Determination of mineral content (namely, Ca, Cd, Cr, Fe, Hg, K, Mg, Mn, Na, Pb and Zn) was performed in a Thermo Scientific ICAP 6000 ICP-Optical Emission Spectroscopy analyzer (Thermo Fisher Scientific Inc., Waltham, MA, USA) and results are summarized in Table 1. No traces of Cd, Cr, Hg, Mn, Pb and Zn were detected in all samples. Sulphate content in κ, ι, λ-CGN and XG was quantitated by CHNS elemental analysis using Flash 2000 Organic elemental analyzer (Thermo Scientific, Waltham, MA, USA) that showed samples contained 52.85 ± 0.29, 79.35 ± 1.00, 61.53 ± 0.88 and 0.00 ± 0.00 mg/gr sulphate, respectively.

**Characterization of stabilizer molecular weights.** Molecular weights of κ-, ι-, and λ- CGN as well as XG were determined by size exclusion chromatography with multi-angle laser light scattering and refractive index detectors (SEC-MALLS-RI system, Postnova analytics, Landsberg, Germany). These analyses were performed on 0.1% (*w*/*v*) of ι- or λ-CGN or 0.05% (*w*/*v*) κ-CGN or XG samples that were pre-dissolved in 0.1M NaNO_3_. Stabilizer samples were filtered using 0.45 µm filters and 100 µL of the filtrate were automatically injected into the SEC-MALLS-RI system. The samples were separated at 35 °C using three packed columns: Ultra-hydrogel 250, 1000 & 2000 (Waters, Milford, MA, USA). SEC eluent was pre-filtered (0.1 µm) 0.1 M NaNO_3_ in DDW used at 0.5 mL/min flow rate. Refractive index (dn/dc) values of 0.12 mL/g were applied to CGN analyses, based on past studies [38,39]. All gathered information was processed using Nova MALS software version 1.5.0.7 (Postnova Analytics, Landsberg, Germany) fitted with the Debye model to calculate molecular weight distributions and results presented as means of duplicates.

**Determination of CGNs and XG Zeta-potentials.** The electrophoretic mobility of κ-, ι-, and λ- CGN and of XG was measured by dynamic laser scattering (Nano-ZS, Malvern Instruments, UK). CGNs or XG were suspended in DDW at pH 7 to obtain samples with similar flow behavior (at final concentrations of 0.1% and 0.01% *w*/*v* CGN or XG, respectively), then balanced using 0.5M HCl or NaOH to obtain samples with pH values of 3–7. The Smoluchowski model was used to interpret DLS data using the Nano-ZS software into zeta-potential values. These experiments were done in duplicates, and each replicate was measured three times.

#### 2.2.2. Preparation and Characterization of CMDs

Chocolate milk drink (CMD) with xanthan gum, κ-, ι- or λ-carrageenan were prepared by dissolving each stabilizer overnight at room temperature in double-distilled water (DDW) pH = 7 to reach a final concentration of 2% *w*/*v*. Subsequently, these were used to produce an initial 2% (*w*/*v*) fat CMD containing 1.192 gr cocoa powder, 5.04 gr sugar and 0.5% *w*/*v* stabilizer [40]. As homogenization is a common processing operation in the production of chocolate milk drinks, the initial drinks were subjected to high-pressure homogenization and pasteurization before being refrigerated before their subsequent analyses (no longer than two days storage).

High pressure homogenization of the CMD formulations was performed in a high pressure homogenizer (model FPG 12800, Stansted Fluid Power Ltd., Harlow, UK) equipped with Y-shaped valve and single piston (9 mL cell) and external heat exchanger set-up to minimize process-induced sample heating. Homogenization was held at a pressure of 55 MPa at Tinlet = 25 °C for one homogenization cycle. Following homogenization, the formulations were heated to 75 °C for 15 s, cooled and refrigerated (4 °C) before their in vitro digestion. Similarly, CMD without any thickener was produced to serve as a control. The pH of all CMD was corrected to pH = 6.5.

**Physicochemical characterization of CMD.** This included determination of CMD viscosity, colloidal particle size and physical stability to separation phenomena, i.e., creaming or cocoa sedimentation. The flow behavior of CMD in the presence or absence of stabilizer was measured using a rheometer (MCR302, Anton Paar, Rhenium, Modiin, Israel) maintained at 37 °C using Peltier elements (P-PTD200 and H-PTD200) (to mimic temperature of human digestion), shear rate of 0.01–100 1/s (using a 50 mm cone plate geometry with 2 degrees cone and a measuring gap of 0.215 mm). The colloidal particle size was determined based on laser scattering (Malvern Mastersizer 3000, Malvern Instruments, UK). The dispersant refractive index was set at 1.330 and particle refractive index was set at 1.34, based on preliminary analysis. Data was processed using the general Fraunhofer model and presented as d4,3 particle distribution and mean diameter. Each measurement was conducted at least in duplicate with every sample measured thrice to ensure consistency and reproducibility. In addition, accelerated physical stability tests were performed on all CMD drinks (LUMisizer, L.U.M. GmbH, Berlin, Germany). Analytical centrifugation cuvettes were filled with 400μL of each sample, then centrifuged for 10.5 h (3000 RPM at 4 °C). Measurement of time and space resolved transmission profiles of samples during centrifugation enabled accelerated comparative analysis of the physical stability.

#### 2.2.3. In Vitro Adult’s or Child’s Digestion of CMD with and without Stabilizers

Evidence show that polysaccharides, such as carrageenan, guar gum, pectin, and carboxymethyl cellulose can modulate digestive processes and mass transfer rates in the gut lumen [13,15,28,29,30]. These experiments interrogated whether and the extent by which stabilizers may affect the in vitro digestive proteolysis of a real food product using a previously described in vitro digestion model (IVD) [37]. Moreover, age-related differences in gastro-intestinal functions were explored via the application of either adult or child gastrointestinal conditions to a dual auto titration unit (Titrando 902, Metrohm, Switzerland) maintained at 37 °C with stirring set at ~250 rpm. TIAMO 2.0 software was used to generate pH gradients in the IVD models based on physiological data described previously [31,37]. During all IVD experiments 1.3 mL aliquots were withdrawn for further analysis (e.g., proteomic analysis): during the gastric phase at 0, 10, 30, 60 and 120 min, and during the duodenal phase at 5, 30 and 60 min. One additional sample from the duodenal stage was collected at 90 min during child in vitro digestion. Inactivation of the enzymes was obtained with 1M ammonium bicarbonate for gastric samples (to increase samples’ pH) or PMSF (0.5 mM) for aspirates collected from the intestinal stage. For subsequent analyses, samples were stored at −20 °C.

**Simulated adult’s digestion.** All IVD experiments relied on the INFOGEST protocol (Minekus et al., 2014). In brief, CMD formulations were mixed (1:1 *v*/*v*) with simulated salivary fluid (SSF) to form a 50 mL oral bolus. Afterwards, 40 mL of the bolus was mixed with 57 mL of preheated simulated gastric fluid (SGF) (pH 1.3, 37 °C). Subsequently, chyme was formed by addition of 3mL of pepsin (2000 U/mL) and 18 µL CaCl_2_ (2 M) into the bioreactor vessel. Gastric chyme pH was adjusted to pH = 4.5 (using 0.1 M NaOH) and computer-controlled gastric pH gradient was initialized (using 0.5M HCl) for a 2 h gastric phase, followed by a 1 h intestinal phase, as detailed before (Shani-Levi et al., 2013). Intestinal phase was initiated by mixing half of the gastric effluent with an equal volume of simulated duodenal fluid (SDF) followed by the sequential addition of trypsin, α-chymotrypsin, bile salts (10 mM) and 6µl CaCl_2_ (2 M). The pH of the intestinal phase was maintained at pH = 6.25 using 0.5 M NH_4_HCO_3_. An additional trypsin and α-chymotrypsin dose was added 10 min after initialization of the intestinal phase (to reach a final concentration of 100 and 50 U/mL, respectively).

**Simulated child’s digestion.** IVD model recreating the conditions of a 2 years old child was performed similarly to the adult IVD model using bio-relevant data of a child’s gut functions, detailed previously [31]. In practice, levels of pepsin, trypsin and chymotrypsin were set at 1760 U/mL, 100 U/mL, and 45 U/mL chymotrypsin in the corresponding digestive phases. Luminal fluid compositions, e.g., simulated gastric fluid, were also adjusted as detailed in Table 2. In addition, the length of the duodenal phase was extended to 90 min (pH was kept at 6.25).

#### 2.2.4. Monitoring Stabilizer Effect on In Vitro Digestive Proteolysis of CMD

This part of the work aimed to investigate the proteolytic breakdown of milk proteins using sodium dodecyl sulfate polyacrylamide gel electrophoresis (SDS-PAGE) and liquid chromatography-mass spectrometry/mass spectrometry (LC-MS/MS) proteomic analyses.

**SDS PAGE analyses.** Qualitative analysis of protein degradation was conducted using 16% Tris-Tricine gels separated at 140 V and 15% acrylamide gels at 180 V, for two different protein ranges: 2–40 kDa and 10–170 kDa, respectively. Coomassie Brilliant blue was used to stain protein bands, followed by distaining with acetic acid/ethanol/DDW and thereupon imaged in a Microtek 9800XL Plus scanner (Microtek, Carson, CA, USA).

**LC-MS/MS peptide profiling.** LC-MS/MS proteomic analyses were conducted on gastric effluents collected after 180 min (G180) of IVD experiments held under a child’s gut conditions. The procedure for sample preparation for MS included denaturation (8 M Urea, 400 mM ammonium bicarbonate and 10 mM Dithiothreitol at 60 °C, 30 min), mixing with Iodoacetamide (8.8 mM) and filtering (centrifugal filter Amicon^®^ Ultra, 0.5 mL, 30 KDa). Samples were desalinated by C18 tips (Ultra-Micro, Harvard) re-suspended in 0.1% Formic acid after being dried. Reversed phase chromatography was used to resolve the peptides on 0.075 × 180-mm fused silica capillaries (J & W) packed with Reprosil (Dr. Maisch GmbH, Germany). Peptide elution gradients were: 60 min linear gradient of 5 to 28%, 15 min gradient of 28 to 95% and 15 min of 95% acetonitrile with 0.1% formic acid in water at flow rates of 0.15 μL/min. these analyses were done on a Q-Exactive plus system (Thermo Scientific, Waltham, MA, USA) operated in a positive mode, repetitively full MS scan followed by collision induces dissociation (HCD) of the 10 most dominant ions selected from the first MS scan. Discoverer 1.4 software with the Sequest algorithm (Thermo Scientific, Waltham, MA, USA) was applied to the data and compared against the Bovine proteome from the Uniprot database. Peptide- and protein-level false discovery rates were filtered to 1% using the target-decoy strategy. The peptide lists were based on a minimum of 2 peptides identified following filtering with high confidence, top rank, mass accuracy.

**Peptidomics analyses.** In order to decipher the bio-relevance of the findings, peptide lists were mined for high homology (>80%) to known bioactive peptides or explored for potentially novel bioactive peptides using a predictive software. First, peptides originating from milk proteins were compared against the Milk Bioactive Peptide Database (MBPDP) http://mbpdb.nws.oregonstate.edu/peptide_search/ [42] to identify homologous sequences and level of homology, as done previously [12,43]. In addition, identified peptide sequences were analyzed in silico using PeptideRanker predictive software [44] in an attempt to identify possible novel bioactive peptides with a bioactivity probability exceeding 80%.

**Data collection and statistics.** All analyses were carried out at least in duplicate, reproducibility verified not to exceed a threshold of 10% difference and data presented as means ± SD. T-test assuming equal variances were accomplished using the Data analysis Toolpak of Microsoft Excel 2013. Principal component analysis was applied using OriginPro 2019 (OriginLAb, Wellesley, MA, USA).

## 3. Results and Discussion

Stabilizers are highly functional and indispensable ingredients in numerous processed foods such as dairy products [45]. Numerous studies elucidate the mechanisms directing the effects of different stabilizers on food properties, e.g., rheology, and shelf life. Yet, there is a need to better understand their roles during human digestion in respect to the possibility of modulating bioaccessibility of macro- and micro-nutrients, as recently shown [28]. This study explores the possible involvement of xanthan gum, κ-,ι-, or λ- carrageenan in modulating the in vitro digestive proteolysis of a model chocolate milk drink (CMD).

### 3.1. Characterization of Stabilizers and CMDs

**Figure 1:**
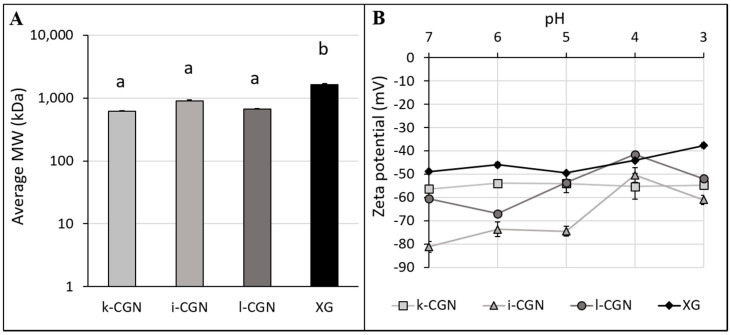
(**A**) Average molecular weight (MW) determined by SEC-RI-MALLS and (**B**) Zeta potential as a function of pH for solutions of κ, ι, λ-CGN (0.1% *w*/*w*) or XG (0.01% *w*/*w*) measured in double-distilled water.

**Figure 2:**
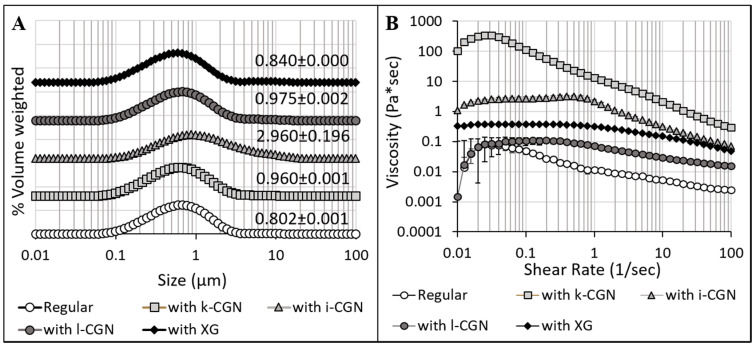
Characteristics of the test chocolate milk drinks; without any stabilizers (regular) and with κ-, ι-, λ-CGN or XG. (**A**) Particle size distribution curves and average d4,3 as well as (**B**) Apparent viscosity measured at 37 °C (*n* = 3).

First, commercial preparations of the stabilizers were obtained and characterized in terms of average MW and zeta-potential (Figure 1) as well as colloidal particle size and flow behavior of the model drinks (Figure 2). Overall, XG had the higher average MW while no significant (*p* < 0.05) differences were noted for the CGN preparations (Figure 1A). Zeta-potential measurements (Figure 1B) indicate that all four stabilizers also have a distinct anionic nature at 3 < pH < 7 (that are relevant to food and the digestive conditions). ι- and λ-CGN was found to have the highest charge levels (−50.46 ± 3.16 mV < zeta potential < −81.22 ± 2.26 mV) as oppose to XG, which had stable yet low levels of charge (−37.68 ± 1.10 mV < zeta potential < −48.86 ± 0.12 mV). Observation seems to be of importance, as electrostatic polysaccharide-protein interactions are elemental in the role of stabilizers in dairy products [46]. Figure 2A shows the effect of the stabilizers on the colloidal particle size distributions of the various test drinks produced, with ι-CGN having the largest colloidal particle sizes. These findings concur with a previous study [47] and show an increase in particle size (d4,3) in the presence of CGN or XG but with no appreciable differences between κ-CGN and λ-CGN. Moreover, this is in line with previous observations that were attributed to the stabilizer interactions with dairy proteins [22,46,48].

**Figure 3:**
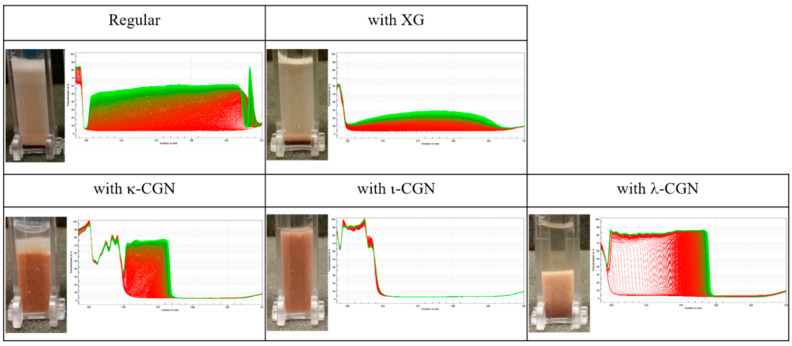
Space and time resolved extinction profiles obtained during analytical centrifugation of chocolate milk drinks in the presence and absence of κ, ι, and λ-CGN or xanthan gum. The Y axis in the graph is the % transmission, and the X axis is the position in nm. Inserts: direct images of test cuvettes after analytical centrifugation.

Characterization of the impact of the stabilizers on the flow behavior of the test drinks (Figure 2B) shows an increase in drink viscosity and the generation of a shear-thinning behavior, in line with previous reports [49,50,51,52,53]. κ-CGN (which has the lowest sulphation degree among the CGNs) had the highest effect on viscosity, followed by ι-CGN, XG and λ-CGN (which has the highest sulphation degree). In the case of CGN, this trend can be attributed to a gelation process [50] in which the more sulphate groups per repeating unit the stronger the CGN-protein interactions. In turn, strong CGN-protein interactions hinder CGN-CGN interactions which are necessary for gelation. λ-CGN had the lowest impact on CMD viscosity, because it can’t form gels as ι-CGN and κ-CGN can [54]. In the case of XG, the flow behavior stems from the combination of high MW and a mild charge. In addition, the effect of the stabilizers was evaluated by accelerated tests in an analytical centrifuge. Figure 3 shows the space and time-resolved extinction profiles of cuvettes filled with the test drinks during centrifugation and corresponding images of cuvettes at the end of the experiments. These findings demonstrate that ι-CGN had the best physical stabilization effect, yielding CMD with the lowest separation compared to all other CMD formulations (including the CMD devoid of stabilizer). Contrary, λ-CGN exacerbated instability phenomena and caused an appreciable sedimentation.

### 3.2. Monitoring Stabilizer Effects on In Vitro Digestive Proteolysis of CMD

**Figure 4:**
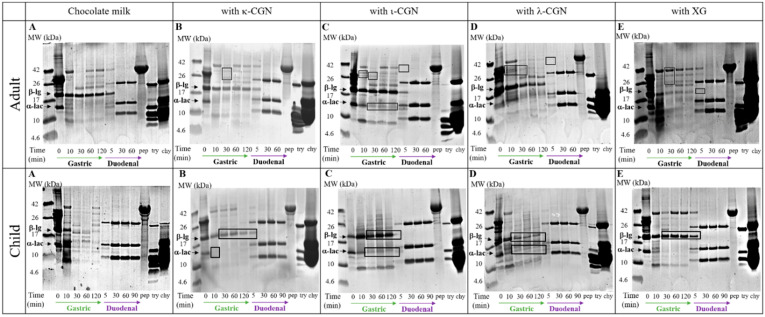
SDS-PAGE analyses of digesta collected during 120 min of gastric and 60/90 min of duodenal in vitro digestion under gastro-intestinal conditions of an adult/child, respectively. (**A**/**F**) Breakdown of CMD (**B**/**G**) Breakdown of CMD in the presence of κ-CGN (**C**/**H**) Breakdown of CMD in the presence of ι-CGN (**D**/**I**) Breakdown of CMD in the presence of λ-CGN (**E**/**J**) Breakdown of CMD in the presence of XG. Pepsin (pep), trypsin (try), chymotrypsin (chy), alpha-lactoglobulin (α-lac) and beta-lactoglobulin (β-lg) are marked in the images.

In light of the varying effects of the stabilizers on the properties and flow behavior of the test CMD, in vitro digestion (IVD) of the CMD samples was performed. SDS-PAGE analyses of gastric and intestinal aspirates collected from a child or adult IVD model provide a qualitative evidence of the differential effects of the stabilizers on the proteolysis of milk proteins (Figure 4 & in Appendix A for protein ranges: 2–40 kDa and 10–170 kDa, respectively). Overall, all stabilizer had varying effects on the extent of digestive proteolysis, which was appreciably noted in changes in the breakdown of α-lg and β-lac and the formation of various patterns of bioaccessible peptides. Moreover, stabilizer effects seem to be selective, with α-lg breakdown being delayed in children while β-lac being the most affected protein in adults. This can be attributed to the differences in ionic composition of the luminal fluids that affect protein conformations and subsequently protein susceptibility to proteases.

In light of an expected high dietary exposure of children to stabilizers, like CGN [36], proteomic analyses focused on aspirates collected from a child IVD model. First, peptide sequences in CMD digesta samples were identified and compared with known milk-derived bioactive peptides. Table 3 lists all peptides that were consistently found in digesta samples and carry homology of at least 80% with bioactive peptides deposited in the MBPDB database [42]. It is important to note that bioactive peptides that were consistently regardless of the CMD formulation have been omitted from Table 3 and gathered in Table A1 in Appendix B. Overall, all stabilizers were found to reduce the total number of bioactive peptides compared to those generated during the digestion of CMD control (without any stabilizer). These findings show that ι-CGN had stronger effect on the total amount of bioaccessible bioactive peptides (inducing the lowest number of bioactive peptides), than λ-CGN followed by κ-CGN/XG; the later two stabilizers having the same effect.

Interestingly, Table 4 summarizes neo-formed bioactive peptides that were only recovered from digesta containing stabilizers. This supports the notion that stabilizer-protein interactions may affect protein conformation and structures that consequently expose different segments to proteolytic enzymes [12]. This may lead to liberation of different bioactive peptides during digestive proteolysis from the same progenitor protein. Another proteomic analysis was used to mine for new bioactive peptides using a predictive software, i.e., PeptideRanker [44]. Table 5. Summarizes bioaccessible peptides recovered from the gastric effluents that were predicted to have a bioactivity probability exceeding 80%. These intriguing peptides should be further explored to ascertain or refute their bioactivity and their possible implications to consumer health and well-being.

**Figure 5:**
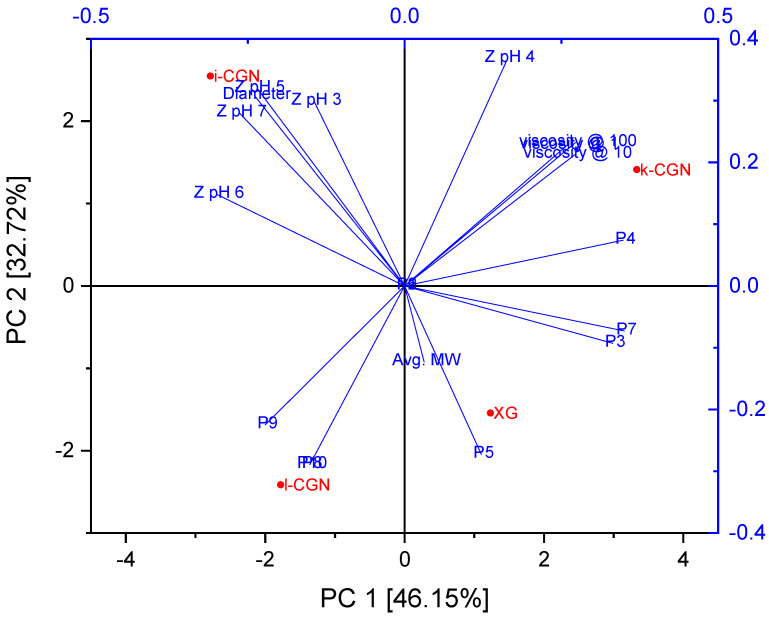
Principal component analysis (PCA) of CMD bioactive peptides recovered in intestinal samples generated during in vitro digestion under a child’s gut conditions as affected by stabilizer type and characteristics. P^#^—numbered bioaccessible bioactive peptide sequence based on the numbering in Table 2 and Table 3; Z pH^#^—Zeta potential measured at pH = ^#^. Diameter = mean d4,3; Viscosity = viscosity measured at a designated shear rate, Avg. MW = Average MW measured by SEC MALS. Viscosity @1, @10 and @ 100 are overlapping in the PCA. Similarly, Diameter overlaps with Z pH 5, P8 overlaps with P10, and P1, P2 and P6 overlap with the origin.

In order to resolve the trends and bio-relevance of the research findings, principal component analysis (PCA) was applied to the data with zeta potentials analyzed as absolute values, for ease of legibility (Figure 5). The PCA analysis cumulative explained total variance of 78.87%. The first principal component (PC1) accounted for 46.15% of the variability in the data set, while PC2 accounted for 32.72% of the variance in the data. This analysis clustered parameters with significant positive correlations in close graphical proximity and negative correlations on opposite graphical quadrants. For example, XG positively affected the generation of peptides P3, P5 and P7 while ι-CGN was negatively correlated to the generation of these peptides. This analysis confirms each stabilizer had unique effects on the proteolysis breakdown patterns with ι-CGN hindering the generation of bioactive peptides to the largest extent, then followed by λ-CGN then by κ-CGN and XG, both with the least effect. Notably, zeta potential at the pH range of CMD production (6 < pH < 7) and colloidal particle size but not viscosity were found to be significant determinants of bioactive peptides generation during digestion. This agrees with previous studies that demonstrated protein/polysaccharide electrostatic interactions dominate the digestive behavior and digestibility of whey proteins [12,70]. Altogether, the macroscopic and non-specific effects of stabilizers on viscosity do not seem to overpower the enzymatic shielding effects arising from macromolecular electrostatic interactions and the binding affinity and avidity between the stabilizers and the various proteins.

To elucidate the underlying mechanism of this phenomenon, UCSF Chimera software (http://www.cgl.ucsf.edu/chimera/1.3/docs/morerefs.html) [71] was used to identify the spatial location of the bioactive peptides that were sensitive to stabilizer addition (Table 2). One would expect that the outer more hydrophilic rim of protein structures would be more accessible to proteases, hence, being more liable to proteolysis over the more hydrophobic or secluded core segments. Our analyses showed that indeed bioaccessible peptides were predominantly located on protein surfaces in more hydrophilic regions. Thus, one can postulate that anionic stabilizers interact with the charged or hydrophilic amino acids on the protein surface thereby limiting their accessibility to proteases. Moreover, this notion is supported by studies that revealed whey gastric proteolysis initiates in cleavage of the protein outer rim where more hydrophilic regions are found [72,73].

## 4. Conclusions

This study sought to extend our understanding of the possible anti-nutritional effects of anionic polysaccharide stabilizers on digestive proteolysis in a realistic model food system of a chocolate milk drink (CMD). Proteomic analyses of effluents from in vitro digestion models demonstrate the heightened sensitivity of children to the addition of stabilizers, as noted in the hindered levels of gastro-intestinal protein breakdown and reduction in number of bioaccessible bioactive peptides. Viscosity and particle sizes did not account for the observed effects of the stabilizers on proteolysis while zeta potential of the stabilizers was clear determinant. The most charged stabilizer, ι-CGN, had the most marked effect on CMD proteolysis probably due to enhanced protein-polysaccharide electrostatic interactions. Thus, supporting the notion that zeta potential is an indicator for the electrostatic polysaccharide-protein interactions that underly the attenuated proteolysis and generation of bioactive peptides. Overall, this demonstrates that anionic polysaccharides have the potential to attenuate the proteolysis of milk proteins of liquid foods in the gut of children. Studies show high correlation between similar in vitro digestion evidence and in vivo findings of macronutrient digestion [74]. Thus, future research should be warranted to further substantiate or refute any possible adverse effects of anionic stabilizers on digestive proteolysis in toddlers and children and other sensitive populations. Moreover, the evidence herein could help improve the design of nourishing and healthier foods as well as provide policy makers with scientific evidence on the possible antinutritional effects of some food stabilizers.

## Figures and Tables

**Table 1:** Elemental analysis of commercial κ-, ι-, λ-Carrageenan (CGN) and Xanthan gum (XG) (mg/gr) determined by ICP.

	Ca	Fe	K	Mg	Na	S
κ-CGN	0.130 ± 0.007	Non detected	62.04 ± 3.573	0.144 ± 0.005	3.850 ± 0.014	46.75 ± 1.040
ι-CGN	0.820 ± 0.031	0.004 ± 0.000	23.31 ± 1.043	0.327 ± 0.068	22.61 ± 1.527	74.85 ± 2.200
λ-CGN	3.905 ± 0.249	Non detected	7.535 ± 0.481	1.913 ± 0.439	17.11 ± 1.514	58.90 ± 0.000
XG	6.820 ± 0.140	Non detected	0.480 ± 0.170	0.070 ± 0.000	3.630 ± 0.130	0.950 ± 0.060

**Table 2:** Composition of simulated digestive fluids [41] used in in vitro digestion experiments mirroring a child’s gut (SGF: simulated gastric fluid, SDF: simulated duodenal fluid (SDF) and SBF: simulated bile fluid).

		Volumes Used from Stock Solutions			
Compound	Stock Solutions [g/L]	Saliva [mL]	SGF [mL]	SDF [mL]	SBF [mL]
KCl	46.72	20.00	56.00	10.80	10.80
KH_2_PO_4_	68.00	40.00	1.800	1.600	35.80
NaHCO_3_	84.00	8.000	26.00	87.00	19.00
NaCl	120.0	2.000	20.00	15.08	16.00
MgCl_2_(H_2_O)_6_	30.00	2.000	4.000	2.200	2.200
NH_4_Cl	27.28	---	2.000	---	---
NaH_2_PO_4_(H_2_O)_2_	166.0	---	---	---	20.00
Urea	22.50	10.00	0.600	4.800	10.40
DDW		918.0	889.6	878.5	885.8
pH adjustment		6.800	1.300	8.100	8.200
CaCl_2_(H_2_O)_2_ 2 M [µL per mL of simulated fluid]		0.273	0.180	1.200	1.850

**Table 3:** Comparison of bioactive peptide sequences to peptides produced during in vitro child’s digestion of chocolate milk drink in the presence of κ, ι and λ-CGN or XG (&κ, &ι, &λ and &X), or without any stabilizer (R). Bold letters emphasize sequence homology between the peptides detected in the digestive effluents and the ones described in the literature. Grey filling appears when the peptide was found in the samples, white—when it was not detected.

Peptide ^#^	Sequence	Known Bioactive Peptide	Activity	%Homology	References	R	&κ	&ι	&λ	&X
P1	AHKALCSEKL	L**AHKALCSEKL**	DPP-IV Inhibitory	90.0	[55]	+				
P2	ALPMHIRL	**ALPMHIR** **ALPMHIR** **ALPMHIR**	stimulates proliferation Reduced vasoconstrictorendothelin-1 release ACE-inhibitory	87.5 87.5 87.5	[56,57,58,59]	+				
P3	KPTPEGDL	L**KPTPEGDL**E L**KPTPEGDL**	DPP-IV Inhibitory DPP-IV Inhibitory	80.0 88.8	[55]	+	+		+	+
P4	LAHKALCSEKL	**LAHKALCSEKL**	DPP-IV Inhibitory	100	[55]	+	+		+	+
P5	NMAINPSKENLCSTF	**NMAINPSKENLCSTF**CK	ACE-inhibitory	88.2	[60]	+	+		+	+
P6	SDIPNPIGSENSEKT	**SDIPNPIGSENSEK**	Antimicrobial	93.3	[61]	+	+			+
P7	VRTPEVDDEAL	**TPEVDDEAL**EK **TPEVDDEAL**EK	DPP-IV Inhibitory Antimicrobial	81.8 81.8	[62,63,64]	+	+		+	+
					Total number	7	5	ND	4	5

**Table 4:** Comparison of bioactive peptide sequences whose generation was induced by the presence of one of the stabilizers during in vitro child’s digestion of the relevant chocolate drinks. Bold letters emphasize sequence homology between the peptides detected in the digestive effluents and the ones described in the literature. Grey filling appears when the peptide was found in the samples, white- when it was not detected.

Peptide ^#^	Sequence	Known Bioactive Peptide	Activity	%Homology	References	R	&κ	&ι	&λ	&X
P8	KIDALNENKV	**IDALNENK** **IDALNENK**	stimulates proliferation antimicrobial	80.0 80.0	[56,62,65]				+	
P9	RPKHPIKHQGLPQEVLNENL	**RPKHPIKHQGLPQEVLNENL**LRFF **RPKHPIKHQGLPQEVLNENL**LRF	Antimicrobial Antimicrobial	83.3 86.9	[66,67,68]			+	+	
P10	RVYVEELKPTPEGDLEIL	**VYVEELKPTPEGDLEIL**LQK	Hypocholesterolemic	85.0	[69]				+	

**Table 5:** Potentially novel bioactive peptides sequences produced during child in vitro digestion of chocolate milk drink in the presence and absence of κ, ι and λ- CGN or XG. Sequences are predicted to have at least 80% probability to be bioactive according to PeptideRanker predictive software tool (Mooney et al., 2012). Grey filling appears when the peptide was found in the samples, white- when it was not detected.

Sequence	Score	R	&κ	&ι	&λ	&X
DRTPPFYCLCPEGF	0.88	+	+	+	+	+
DSWPCVMGR	0.92					+
FGKNGKNCPDKFCL	0.88	+	+	+	+	+
FGSPPGQRDLL	0.80	+	+	+	+	+
FSQSCAPGADPKSRL	0.82	+	+	+	+	+
GGVSLPEWVCTTF	0.84	+	+	+	+	+
VRETCGCCDCEKRCGAL	0.82	+	+	+	+	+

**Figure A1:**
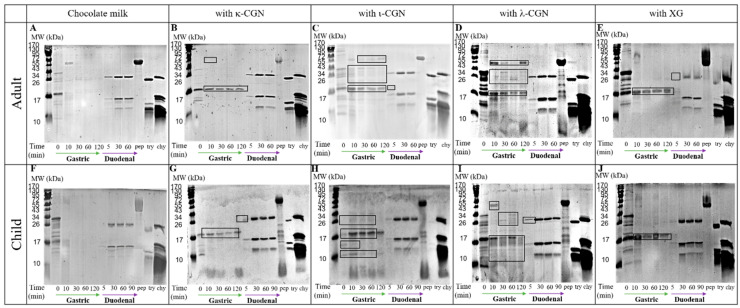
SDS-PAGE analyses (in the range of 10–170kDa) of digesta collected during 120 min of gastric and 60/90 min of duodenal in vitro digestion under gastro-intestinal conditions of an adult/child, respectively. (**A**/**F**) Breakdown of CMD (**B**/**G**) Breakdown of CMD in the presence of κ-CGN (**C**/**H**) Breakdown of CMD in the presence of ι-CGN (**D**/**I**) Breakdown of CMD in the presence of ι-CGN (**E**/**J**) Breakdown of CMD in the presence of XG. Pepsin (pep), trypsin (try), chymotrypsin (chy), alpha-lactoglobulin (α-lac) and beta-lactoglobulin (β-lg) are marked in the images.

## Appendix B

**Table A1:** Comparison of bioactive peptide sequences to peptides produced during in vitro digestion of chocolate milk drink in the presence and absence of κ, ι and λ- CGN or XG. Bold letters emphasize sequence homology between the peptides detected in the digestive effluents and the ones described in the literature. Grey filling appears when the peptide was found in the samples, white—when it was not detected.

Sequence	Known Bioactive Peptide	Activity	Homology %	References	R	&κ	&ι	&λ	&X
ARHPHPHLSF	**HPHPHLSF**	ACE-inhibitory	80	[58,75]	+	+	+	+	+
AVPYPQRDMPIQAF	**VPYPQRDMPIQAF**L	Antimicrobial	92.9	[68]	+	+	+	+	+
DELQDKIHPF	TE**DELQDKIHPF**	Antimicrobial	83.3	[76]	+	+	+	+	+
DIQKVAGTW	**IQKVAGTW**	DPP-IV Inhibitory, ACE-inhibitory, Antimicrobial, ACE-inhibitory	88.9	[76,77]	+	+	+	+	+
	**IQKVAGTW**		88.9						
	GL**DIQKVAGT**		80						
	L**DIQKVAGT**W		80						
ELKPTPEGDL	**LKPTPEGDL**E	DPP-IV Inhibitory DPP-IV Inhibitory	90	[55]	+	+	+	+	+
	**LKPTPEGDL**		90						
FSDKIAKY	**FSDKIAK**	Antimicrobial ACE-inhibitory	87.5	[78,79]	+	+	+	+	+
	**FSDKIAK**		87.5						
FTKKTKLTEEEKNRL	**TKKTKLTEEEKNRL**	Antimicrobial	93.3	[80]	+	+	+	+	+
IIAEKTKIPAVF	**IIAEKTKIPAVF**	Antimicrobial	100	[76]	+	+	+	+	+
IQPKTKVIPYVRYL	W**IQPKTKVIPYVRYL**	Antimicrobial Antimicrobial	93.3	[80]	+	+	+	+	+
	**IQPKTKVIPYVR**		85.7						
KTKLTEEEKNRL	TK**KTKLTEEEKNRL**	Antimicrobial	85.7	[80]	+	+	+	+	+
KTKLTEEEKNRLNF	TK**KTKLTEEEKNRL**	Antimicrobial	85.7	[80]	+	+	+	+	+
LKPTPEGDL	**LKPTPEGDL**E	DPP-IV Inhibitory DPP-IV Inhibitory	90	[55]	+	+	+	+	+
	**LKPTPEGDL**		100						
LVYPFPGPIPNSL	**LVYPFPGPIPNSL**PQN **LVYPFPGPIPNSL**PQ	ACE-inhibitory ACE-inhibitory	81.3	[81,82]	+	+	+	+	+
			86.7						
MAIPPKKNQDKTEIPTINT	**MAIPPKKNQDKTEIPTINT MAIPPKKDQDKTE**VPAINT	Antimicrobial Antimicrobial	100	[76,83]	+	+	+	+	+
			68.4						
MAIPPKKNQDKTEIPTINT	**MAIPPKKNQDKTEIPTINT MAIPPKKDQDKTE**VPAINT	Antimicrobial Antimicrobial	100	[76,83]	+	+	+	+	+
			68.4						
PQNIPPLTQT	L**PQNIPPLT**	DPP-IV Inhibitory ACE-inhibitory	80	[84,85]	+	+	+	+	+
	**NIPPLTQT**PV		80						
PQNIPPLTQTP	**NIPPLTQTP**V	ACE-inhibitory	81.8	[84]	+	+	+	+	+
PTVMFPPQSVL	S**PTVMFPPQSVL**	DPP-IV Inhibitory	91.7	[86]	+	+	+	+	+
PVLGPVRGPF	E**PVLGPVRGP**	Cytomodulatory ACE-inhibitory ACE-inhibitory	90	[87,88,89,90]	+	+	+	+	+
	**VLGPVRGPF**P		90						
	E**PVLGPVRGPF**P		83.3						
PVLGPVRGPFPIIV	YQE**PVLGPVRGPFPIIV**	Immunomodulatory Antithrombin Antimicrobial ACE-inhibitory Antimicrobial ACE-inhibitory	82.4	[66,76,81,91,92,93]	+	+	+	+	+
	YQE**PVLGPVRGPFPIIV**		82.4						
	YQE**PVLGPVRGPFPIIV**		82.4						
	YQE**PVLGPVRGPFPIIV**		82.4						
	YQE**PVLGPVRGPFPI**		80						
	QE**PVLGPVRGPFPIIV**		87.5						
RHPHPHLSF	**HPHPHLSF**	ACE-inhibitory	88.9	[58,75]	+	+	+	+	+
RPKHPIKHQGL	**RPKHPIKHQ**	ACE-inhibitory	81.8	[94]	+	+	+	+	+
SDIPNPIGSENSE	**SDIPNPIGSENSE**K	Antimicrobial	92.9	[61]	+	+	+	+	+
SDIPNPIGSENSEK	**SDIPNPIGSENSEK**	Antimicrobial	100	[61]	+	+	+	+	+
SDIPNPIGSENSEKTTM	**SDIPNPIGSENSEK**	Antimicrobial	82.4	[61]	+	+	+	+	+
SDIPNPIGSENSEKTTM	**SDIPNPIGSENSEK**	Antimicrobial	82.4	[61]	+	+	+	+	+
SDKIAKY	F**SDKIAK**	Antimicrobial ACE-inhibitory	85.7	[78,79]	+	+	+	+	+
	F**SDKIAK**		85.7						
SLSQSKVLPVPQ	**SQSKVLPVPQ**	ACE-inhibitory	83.3	[87]	+	+	+	+	+
SQSKVLPVPQK	**SQSKVLPVPQ**	ACE-inhibitory ACE-inhibitory	90.9	[87,93]	+	+	+	+	+
	**SKVLPVPQ**		72.7						
TKKTKLTEEEKNRL	**TKKTKLTEEEKNRL**	Antimicrobial	100	[80]	+	+	+	+	+
TKKTKLTEEEKNRLNF	**TKKTKLTEEEKNRL**	Antimicrobial	87.5	[80]	+	+	+	+	+
VEELKPTPEGDL	**VEELKPTPEG**NLE	Antimicrobial	76.9	[76]	+	+	+	+	+
VLDTDYKK	**VLDTDYK**	ACE-inhibitory	87.5	[81]	+	+	+	+	+
VYPFPGPIPN	**VYPFPGPIP**	prolyl endopeptidase-inhibitory PEP-inhibitory prolyl endopeptidase-inhibitory PEP-inhibitory DPP-IV Inhibitory ACE-inhibitory Antioxidant ACE-inhibitory ACE-inhibitory ACE-inhibitory ACE-inhibitory	90	[86,94,95,96,97,98,99,100]	+	+	+	+	+
	**VYPFPGPIP**		90						
	**YPFPGPIPN**		90						
	**YPFPGPIPN**		90						
	**VYPFPGPIPN**		100						
	**VYPFPGPIPN**		100						
	L**VYPFTGPIPN**		90.9						
	L**VYPFPGPIP**		90						
	**VYPFPGPI**		80						
	**VYPFPGPI**		80						
	**YPFPGPIPN**		90						
	**YPFPGPIPN**		90						
	**VYPFPGPIPN**		100						
	**VYPFPGPIPN**		100						
	L**VYPFTGPIPN**		90.9						
	L**VYPFPGPIP**								
	L**VYPFPGPI**								
VYPFPGPIPNS	**VYPFPGPIP**	prolyl endopeptidase-inhibitory PEP-inhibitory DPP-IV Inhibitory ACE-inhibitory Antioxidant ACE-inhibitory ACE-inhibitory ACE-inhibitory	81.8	[94,95,96,99,100,101]	+	+	+	+	+
	**VYPFPGPIP**		81.8						
	**YPFPGPIPN**		81.8						
	**YPFPGPIPN**		81.8						
	**VYPFPGPIPN**		90.9						
	**VYPFPGPIPN**		90.9						
	L**VYPFTGPIPN**		90.9						
	L**VYPFPGPIP**		81.8						
VYPFPGPIPNSL	**VYPFPGPIPN**	Antioxidant ACE-inhibitory ACE-inhibitory	83.3	[82,96]	+	+	+	+	+
	**VYPFPGPIPN**		83.3						
	L**VYPFPGPIPNSL**PQ		80						
YQEPVLGPVRGPF	**YQEPVLGPVRGPF**PI	Antimicrobial ACE-inhibitory ACE-inhibitory	86.7	[66,76,87,102]	+	+	+	+	+
	**YQEPVLGPVRG**		84.6						
	**EPVLGPVRGPF**P		84.6						
YQEPVLGPVRGPFPIIV	**YQEPVLGPVRGPFPIIV**	Immunomodulatory Antithrombin Antimicrobial ACE-inhibitory Antimicrobial ACE-inhibitory ACE-inhibitory	100	[66,76,91,92,93,103]	+	+	+	+	+
	**YQEPVLGPVRGPFPIIV**		100						
	**YQEPVLGPVRGPFPIIV**		100						
	**YQEPVLGPVRGPFPIIV**		100						
	**YQEPVLGPVRGPFPI**		88.2						
	**QEPVLGPVRGPFPIIV**		94.1						
	LL**YQEPVLGPVRGPFPIIV**		89.4						
YQKFPQY	**YQKFPQY**	Antioxidant ACE-inhibitory	100	[104,105]	+	+	+	+	+
	**YQKFPQY**		100						
YQKFPQYL	**YQKFPQ**Y	Antioxidant ACE-inhibitory	87.5	[104,105]	+	+	+	+	+
	**YQKFPQ**Y		87.5						
YQQKPVAL	**YYQQKPVA**	Antimicrobial	87.5	[78]	+	+	+	+	+
YVEELKPTPEGDL	**VEELKPTPEG**NLE	Antimicrobial	76.9	[76]	+	+	+	+	+
